# Association between miR-31-3p expression and cetuximab efficacy in patients with KRAS wild-type metastatic colorectal cancer: a post-hoc analysis of the New EPOC trial

**DOI:** 10.18632/oncotarget.21291

**Published:** 2017-09-27

**Authors:** Siân Pugh, Raphaële Thiébaut, John Bridgewater, Marie-Lise Grisoni, Karwan Moutasim, Francis Rousseau, Gareth J. Thomas, Gareth Griffiths, François Liebaert, John Primrose, Pierre Laurent-Puig

**Affiliations:** ^1^ University Surgery, Cancer Sciences, University of Southampton, Southampton, United Kingdom; ^2^ IntegraGen SA, Evry, France; ^3^ UCL Cancer Institute, London, United Kingdom; ^4^ Cancer Sciences Division, University of Southampton, Southampton, United Kingdom; ^5^ Southampton Clinical Trials Unit, University of Southampton, Southampton, United Kingdom; ^6^ UMR-S1147, University Paris Descartes, Paris, France; ^7^ Assistance Publique-Hôpitaux de Paris, Hôpital Georges Pompidou, Paris, France

**Keywords:** metastatic colorectal cancer, biomarker, miR-31-3p, anti-EGFR, cetuximab

## Abstract

**Background:**

High miR-31-3p expression is associated with inferior outcomes in KRAS wild-type (WT) advanced colorectal cancer patients treated with anti-EGFR therapy. This study evaluated miR-31-3p expression in patients with operable colorectal liver metastases (LM) enrolled in the New EPOC study.

**Methods:**

MiR-31-3p expression was measured in primary tumors (PT) from 149 KRAS WT patients including 71 receiving chemotherapy alone (CT) and 78 receiving chemotherapy plus cetuximab (CTX). Each treatment arm was split into tertiles based on miR-31-3p expression levels. MiR-31-3p expression was also measured in LM from 94 patients with tumor tissue available.

**Results:**

The median progression-free survival for the combined populations with mid or high miR-31-3p expression was shorter in the CTX versus the CT arm (26.7 months versus 12.3 months, HR=2.28 95%CI 1.27; 4.09 p=0.006). Low miR-31-3p expressers had similar outcomes irrespective of treatment (HR=1.06 95%CI 0.43; 2.61 p=0.9). MiR-31-3p expression was correlated between paired PT and LM samples in the CT group but not in the CTX group.

**Conclusions:**

Patients with low miR-31-3p expression in the New EPOC study were not harmed by the addition of cetuximab. This supports miR-31-3p as a promising predictive biomarker for anti-EGFR therapy in KRAS WT advanced colorectal cancer.

## INTRODUCTION

There have been significant improvements in the management of colorectal cancer over the last decade involving increasingly sophisticated combinations of chemotherapy, targeted agents, and surgery. This has included the use of anti-epidermal growth factor receptor (EGFR) antibodies such as cetuximab. Following several studies demonstrating the benefit of cetuximab as a single agent or in combination with chemotherapy for advanced colorectal cancer, [1-4] its role was evaluated as an adjunct to chemotherapy and surgery for operable colorectal liver metastases in the New EPOC study. [5] Unexpectedly, this resulted in a shorter progression-free survival for patients treated with cetuximab in contrast to previous data associated with its use in inoperable disease. This effect persisted despite restriction of patients to an all RAS subgroup, suggesting that additional biomarkers linked with treatment efficacy have yet to be identified [6].

MicroRNAs (miRNA) are small non-coding RNA molecules that play a key role in the regulation of intracellular processes through post-transcriptional regulation of gene expression. The stable presence of miRNAs in formalin-fixed paraffin-embedded (FFPE) tissues and their involvement in multiple physiological pathways and pathologies suggests an opportunity to identify new biomarkers for multiple diseases. [7-11] MicroRNAs controlling expression of oncogenes and tumor suppressor genes have been shown to be frequently deregulated in cancer cells [7-9] suggesting potential biomarker candidates for precision oncology.

A miRNA which has both oncogenic and tumor suppressive functional roles frequently deregulated in a variety of cancers is mir-31. [12] In colorectal cancer, mir-31 is typically overexpressed with high expression correlating with advanced disease. [13-15] Functional studies have shown mir-31 has pleiotropic activity, promoting colorectal cancer progression through enhanced migration and invasion. [16, 17] While the most frequently identified mature sequence of mir-31 identified in the literature is miR-31-5p, the other mature sequence of mir-31, miR-31-3p, has been reported to be associated with outcomes for patients treated with anti-EGFR therapy. [18-20] As a follow-up to these findings, the present study sought to evaluate whether expression of miR-31-3p could predict outcomes in the setting of operable colorectal liver metastasis in patients treated with chemotherapy plus cetuximab enrolled in the New EPOC study.

## RESULTS

**Figure 1:**
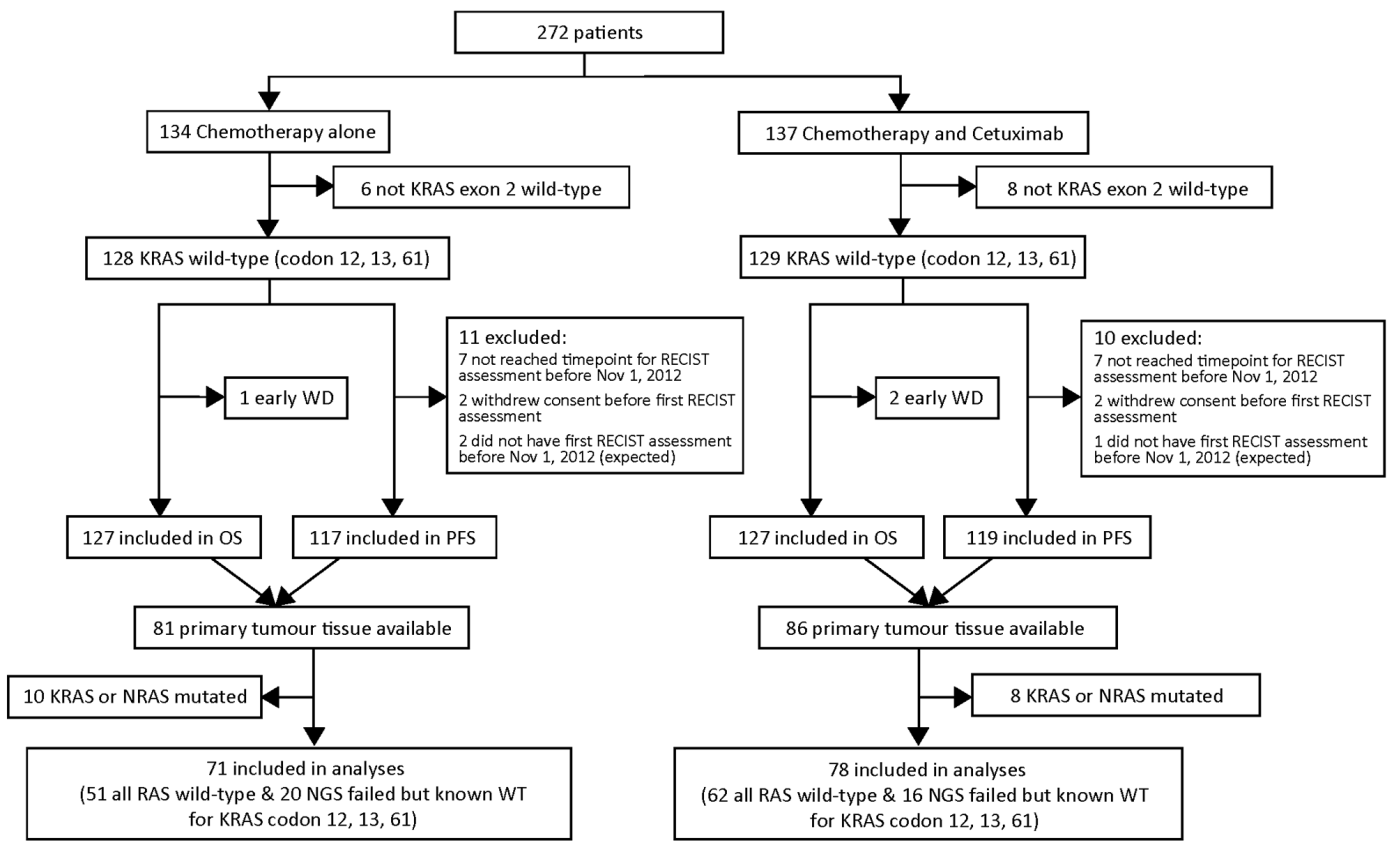
Flow diagram for patients included in study

The modified ITT population (mITT) was composed of 149 patients, 78 patients in a chemotherapy plus cetuximab arm and 71 patients in a chemotherapy alone arm (Figure 1). One hundred and thirteen of these patients were confirmed all RAS WT. Thirty-six patients in the mITT population had incomplete RAS mutational status by NGS but were known to be KRAS WT (codons 12, 13, and 61) based on pyrosequencing results obtained during the original New EPOC study.

### Patient baseline characteristics in mITT and association with miR-31-3p expression

In the mITT population, baseline characteristics were well balanced between treatment arms (Table 1) and between miR-31-3p expression tertiles (Table 2) with the exception of BRAF mutational status, which was more frequent in the high tertile group (6/7 BRAF mutations were in patients with high miR-31-3p expression, p=0.017).

**Table 1:** Baseline characteristics in the mITT population for each treatment arm

	Chemotherapy n = 71	Chemotherapy plus cetuximab n = 78
**Sex**		
F	25 (35.2%)	22 (28.2%)
M	46 (64.8%)	56 (71.8%)
**Age**		
[0-60]	20 (28.2%)	19 (24.4%)
[60-70]	34 (47.9%)	39 (50%)
[70+]	17 (23.9%)	20 (25.6%)
**Chemotherapy regimen**		
CapOx	15 (21.4%)	18 (23.4%)
FOLFIRI	6 (8.6%)	12 (15.6%)
FOLFOX	49 (70%)	47 (61%)
**BRAF status**		
WT/Unknown	68 (95.8%)	73 (94.8%)
Mutated	3 (4.2%)	4 (5.2%)
**Primary tumor location**		
Left sided	59 (83.1%)	59 (75.6%)
Right sided	12 (16.9%)	19 (24.4%)
**Presentation of metastases**		
Non-synchronous	33 (46.5%)	24 (30.8%)
Synchronous	38 (53.5%)	54 (69.2%)
**Number of liver metastases**		
< 4	57 (81.4%)	58 (74.4%)
≥ 4	13 (18.6%)	20 (25.6%)
**ECOG performance status**		
0	51 (71.8%)	54 (69.2%)
1	20 (28.2%)	22 (28.2%)
2	0 (0%)	2 (2.6%)
**miR tertiles**		
Low	23 (32.4%)	27 (34.6%)
Intermediate	20 (28.2%)	29 (37.2%)
High	28 (39.4%)	22 (28.2%)

**Table 2:** Baseline characteristics in the mITT population for each miR-31-3p tertile

	Low n = 50	Intermediate n = 49	High n = 50	p-value
**Sex**				
F	13 (26%)	19 (38.8%)	15 (30%)	0.401
M	37 (74%)	30 (61.2%)	35 (70%)	
**Age**				
[0-60]	11 (22%)	12 (24.5%)	16 (32%)	0.815
[60-70]	27 (54%)	24 (49%)	22 (44%)	
[70+]	12 (24%)	13 (26.5%)	12 (24%)	
**Chemotherapy regimen**				
CapOx	10 (20.4%)	10 (20.4%)	13 (26.5%)	0.439
FOLFIRI	3 (6.1%)	8 (16.3%)	7 (14.3%)	
FOLFOX	36 (73.5%)	31 (63.3%)	29 (59.2%)	
**BRAF status**				
WT/Unknown	49 (98%)	48 (100%)	44 (88%)	0.017
Mutated	1 (2%)	0 (0%)	6 (12%)	
**Primary tumor location**				
Left+rectum	43 (86%)	39 (79.6%)	36 (72%)	0.229
Right	7 (14%)	10 (20.4%)	14 (28%)	
**Presentation of metastases**				
Non-synchronous	18 (36%)	19 (38.8%)	20 (40%)	0.933
Synchronous	32 (64%)	30 (61.2%)	30 (60%)	
**Number of liver metastases**				
< 4	37 (75.5%)	38 (77.6%)	40 (80%)	0.855
≥ 4	12 (24.5%)	11 (22.5%)	10 (20%)	
**ECOG performance status**				
0	34 (68%)	34 (69.4%)	37 (74%)	0.850
1	15 (30%)	15 (30.6%)	12 (24%)	
2	1 (2%)	0 (0%)	1 (2%)	

### Patient clinical outcomes in mITT population

Despite a high percentage of censored patients (52% for progression-free survival and 82% for overall survival) progression-free survival was significantly shorter in the chemotherapy plus cetuximab arm versus the chemotherapy alone arm with a median of 14.5 months versus 24.2 months (HR=1.8 [1.1 ; 2.9], p=0.02). The same was observed for overall survival (HR=2.2 [1.0 ; 4.9], p=0.047). The above aligns with previously published results from the New EPOC interim analysis. [20]

### miR-31-3p as a prognostic marker in patients treated with chemotherapy plus cetuximab

**Figure 2:**
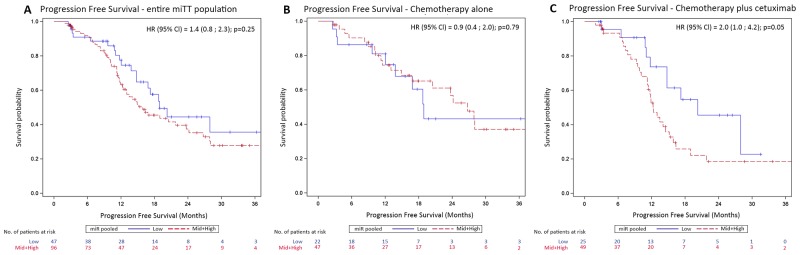
Kaplan-Meier curves of progression-free survival (PFS) according to miR-31-3p expression level groups **(A)** PFS for the entire miTT population; **(B)** PFS for the chemotherapy alone treatment arm **(C)** PFS for the chemotherapy plus cetuximab treatment arm.

Progression-free survival was not significantly different between the miR-31-3p expression subgroups in the whole mITT population (Figure 2A) with a median progression free survival of 18.0 months in the low expression subgroup versus 15.9 months in the combined mid plus high expression subgroup (HR=1.4 [0.8 ; 2.3] p=0.25). In the chemotherapy alone arm (Figure 2B), progression-free survival was not statistically different between the miR-31-3p expression subgroups (HR=0.9 [0.4 ; 2.0], p=0.79). In contrast, patients in the chemotherapy plus cetuximab arm (Figure 2C) with low miR-31-3p expression had a longer progression-free survival compared to patients in the combined mid plus high expression subgroup (median of 20.3 months versus 12.3 months, HR=2.0 [1.0 ; 4.2], p=0.049). Overall survival was not significantly different between the miR-31-3p expression groups in the whole mITT population (HR=0.9 [0.4 ; 2.1], p=0.86), in the chemotherapy alone arm (HR=0.7 [0.2 ; 2.9], p=0.62), or in the chemotherapy plus cetuximab arm (HR=1.1 [0.4 ; 3.0], p=0.85).

Objective response rates did not significantly vary according to miR-31-3p expression in the whole mITT population with 34 patients (68%) achieving complete or partial response out of the 50 patients with low miR-31-3p expression and 62 patients (63%) out of the 99 in the mid plus high miR-31-3p expression subgroup, (p=0.59). There was also no difference in objective response rate when analyzed by treatment arm. For the chemotherapy alone arm, 15 of the 23 patients (65%) with low miR-31-3p expression and 27 of the 48 patients (56%) in the mid plus high miR-31-3p expression subgroup achieved complete or partial responses (p=0.61). For the chemotherapy plus cetuximab arm, 19 of the 27 patients (70%) with low miR-31-3p expression and 35 of the 51 (69%) with mid or high miR-31-3p expression had a complete or partial response (p=1.0).

### miR-31-3p as predictive marker of cetuximab efficacy on survival outcomes

**Figure 3:**
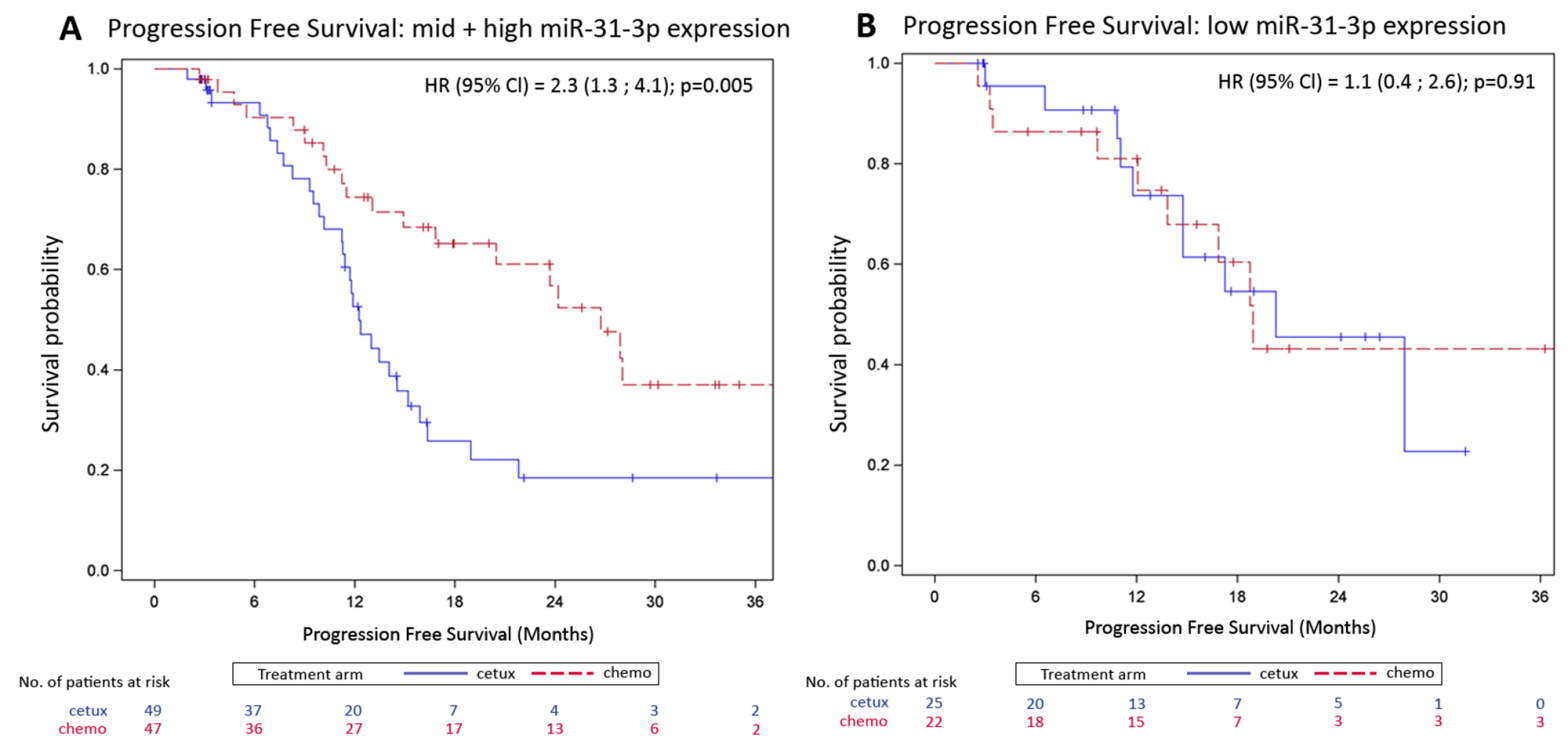
Kaplan-Meier curves of progression-free survival (PFS) by treatment arm **(A)** OS for the mid plus high miR-31-3p expressers; **(B)** OS for low miR-31-3p expressers.

**Figure 4:**
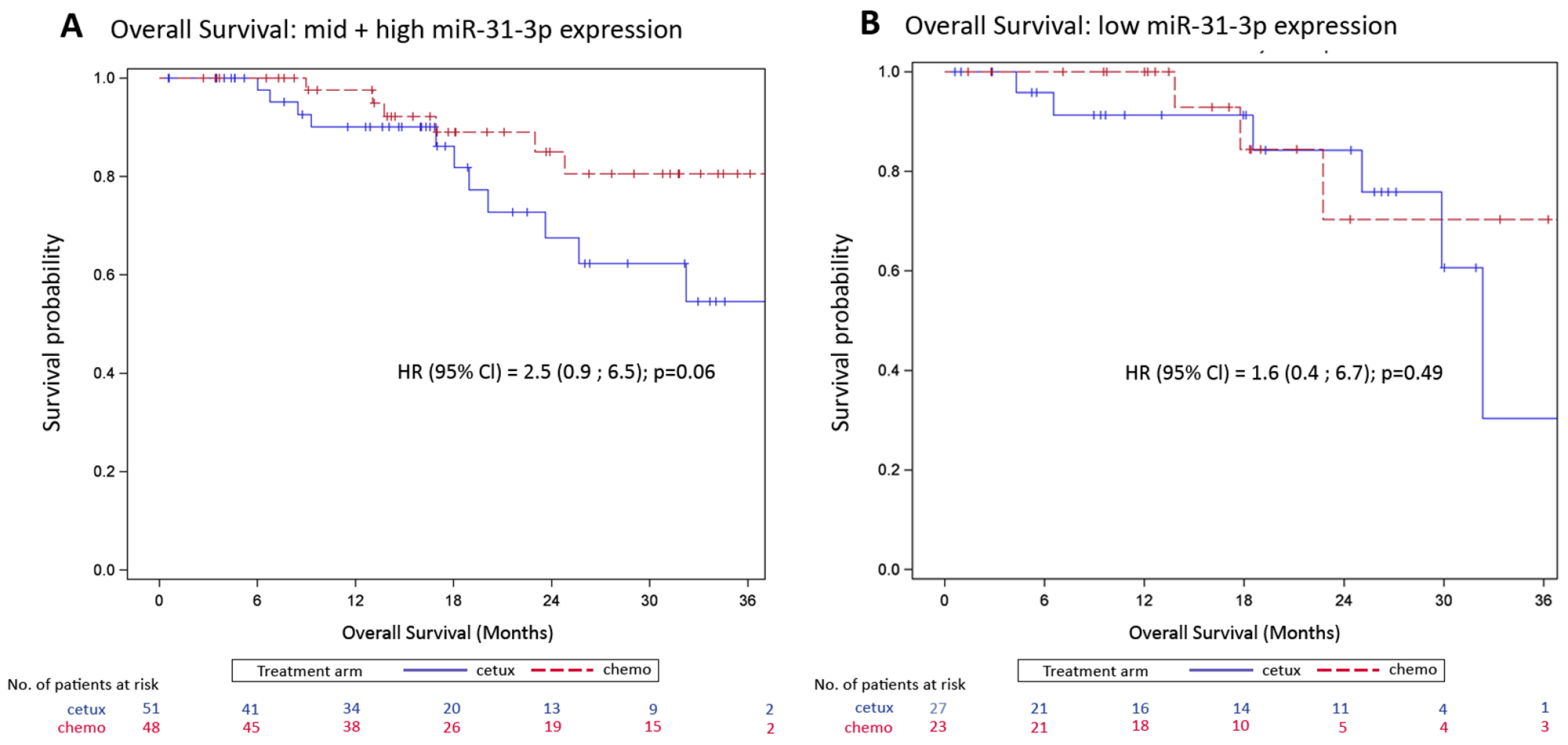
Kaplan-Meier curves of overall survival (OS) by treatment arm **(A)** OS for the mid plus high miR-31-3p expressers; **(B)** OS for low miR-31-3p expressers.

Patients in the mid plus high miR-31-3p expression group treated with cetuximab had a shorter progression free survival compared to patients treated with chemotherapy alone (median progression free survival 12.3 versus 26.7 months respectively; HR=2.3 [1.3 ; 4.1], p=0.005). This difference was not observed in low expressers (median progression free survival 20.3 months versus 18.9 months respectively; HR=1.1 [0.4 ; 2.6] p=0.91) (Figure 3). Patients in the mid plus high miR-31-3p expression group treated with cetuximab also had a non-significantly shorter overall survival compared to patients treated with chemotherapy alone (HR=2.5 [0.9; 6.5], p=0.06). This difference was not observed in low expressers (HR=1.6 [0.4 ; 6.7] p=0.49) (Figure 4). The treatment-miR-31-3p expression group interaction test was not significant (p=0.16).

Objective response rates were not significantly different according to treatment arm. For the mid plus high miR-31-3p expression subgroup, 35 of the 51 patients (68%) in the chemotherapy plus cetuximab arm, and 27 of the 48 patients (56%) in the chemotherapy alone arm achieved either a partial or complete response; p=0.22. For the low expressers subgroup, 19 of the 27 patients (70%) in the chemotherapy plus cetuximab arm and 15 of the 23 patients (65%) in the chemotherapy alone arm achieved either a partial or complete response; p=0.77.

### Multivariate analyses

Consistent with the univariate analysis results, multivariate analyses adjusted for confounding factors showed that miR-31-3p expression group was not a significant predictive factor for progression free survival in the overall population or in the chemotherapy alone arm, but remained significant in the cetuximab treated population (low versus mid-high, HR = 2.1 [1.0; 4.4], p=0.05).

### Comparison of miR-31-3p expression between metastases and primary tumors

**Figure 5:**
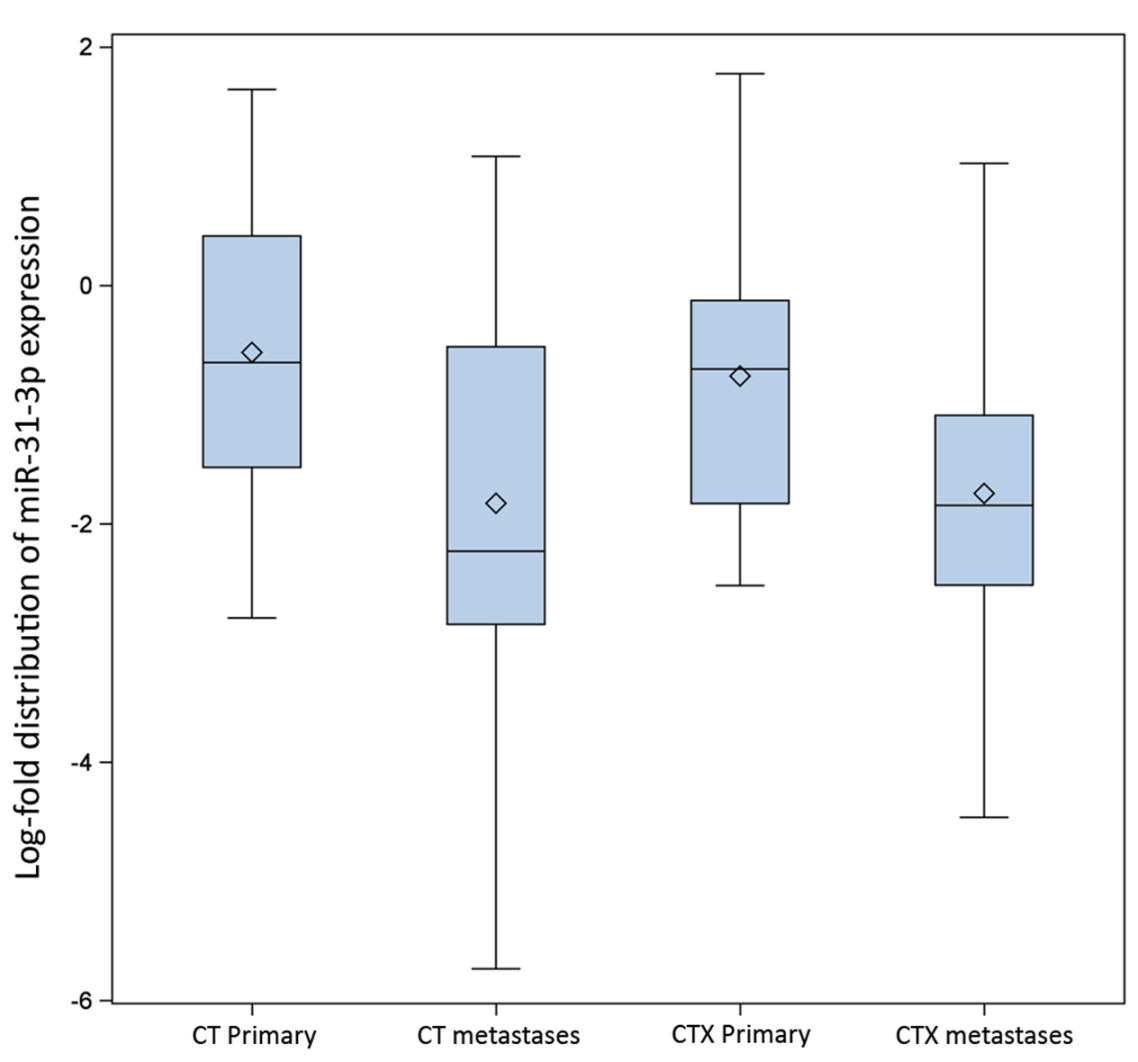
Distribution of miR-31-3p expression (logarithmic scale) by treatment arm, in primary tumor and liver metastases (CT = chemotherapy alone treatment arm; CTX = chemotherapy + chemotherapy treatment arm).

An analysis of miR-31-3p expression in liver metastases collected post neoadjuvant treatment versus primary tumors collected at baseline was performed using 94 paired samples of primary tumors and liver metastases. MiR-31-3p expression was significantly lower in metastases compared to primary tumors with a mean (std) of log-fold of -0.66 (1.18) for primary tumors versus -1.79 (1.37) for liver metastases, p<0.001. Expression levels did not significantly differ between treatment arms either in primary tumors (-0.81 [1.11] for chemotherapy plus cetuximab versus -0.87 [1.18] for chemotherapy alone, p=0.45) or in liver metastases (-1.68 [1.28] for chemotherapy plus cetuximab versus -1.96 [1.43] for chemotherapy alone, p=0.76). The absolute change of expression between treatment arms was not significantly different (0.87 [1.32] for chemotherapy plus cetuximab versus 1.08 [1.62] for chemotherapy alone, p=0.36) (Figure 5).

**Figure 6:**
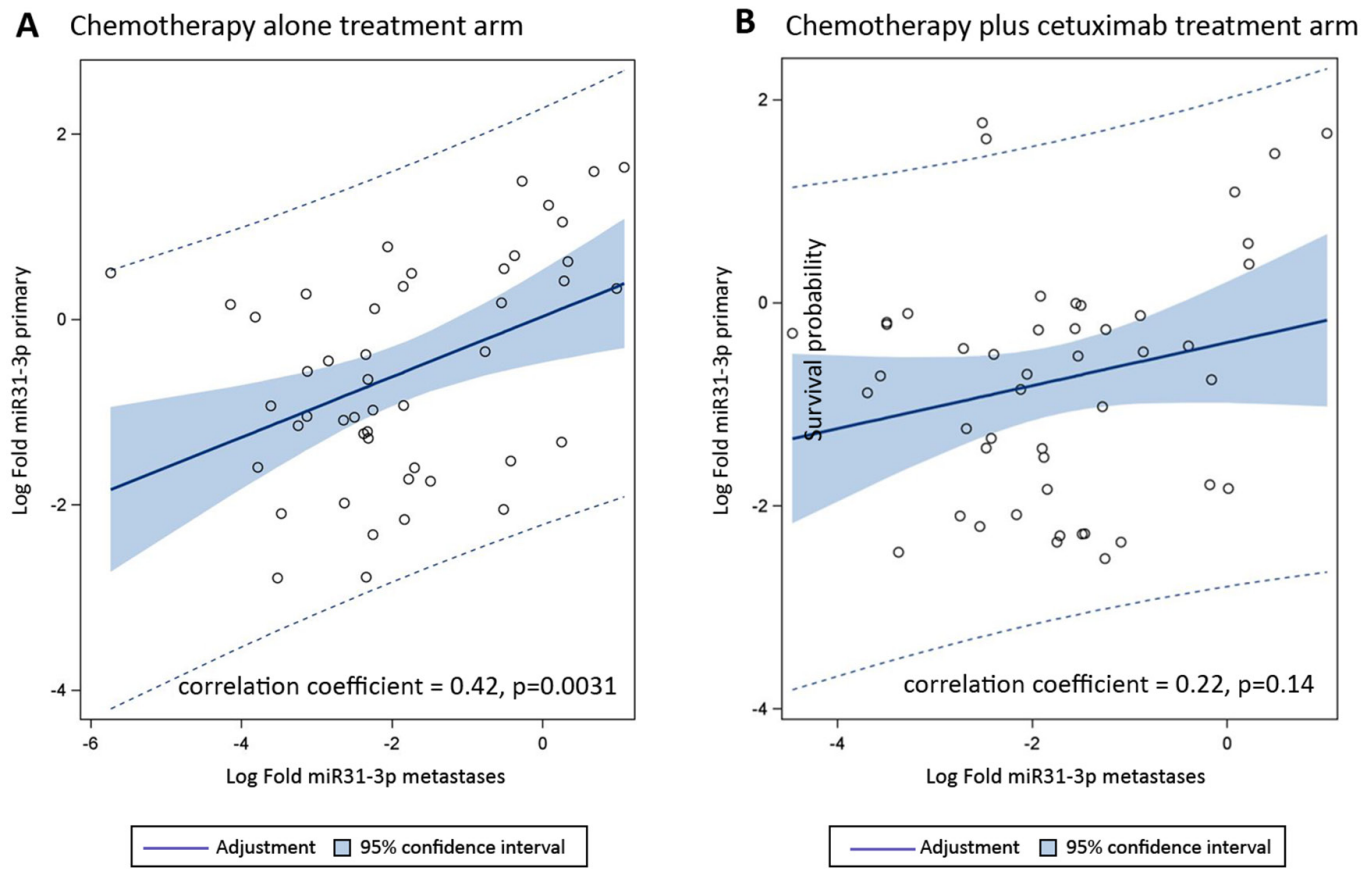
Correlation of miR-31-3p expression (log-transformed) between paired primary tumors and liver metastases **(A)** chemotherapy alone treatment arm; **(B)** chemotherapy + cetuximab treatment arm.

In the 47 paired samples from the chemotherapy alone arm, miR-31-3p expression levels in primary tumors and corresponding metastases were highly correlated with a correlation coefficient of 0.42; p=0.0031. In contrast, for the 47 paired samples from the chemotherapy plus cetuximab arm, a lower, non-statistically significant correlation was observed (correlation coefficient = 0.22, p=0.14) (Figure 6A and 6B).

## DISCUSSION

The New EPOC study demonstrated a shorter progression-free survival with the addition of cetuximab to chemotherapy for patients with operable colorectal liver metastases. [5] While it is a reminder that the consequences of combining treatment strategies can be unpredictable, it does afford a unique opportunity to increase our understanding of tumor responses to treatment with anti-EGFR therapy. Not only can putative predictive biomarkers be assessed in the “pre-treatment” primary tumor tissue, but the additional availability of “post-treatment” resected liver metastases enables paired analyses to aid a mechanistic dissection of the effect of cetuximab.

In our study we observed that in the subgroup of patients treated with cetuximab, patients in the combined mid plus high miR-31-3p expression group had a shorter progression free survival in comparison to patients treated with low miR-31-3p expression. By contrast in the chemotherapy alone arm, progression free survival was similar regardless of miR-31-3p expression level. We also observed that patients in the mid plus high miR-31-3p expression group had a shorter progression free survival and shorter overall survival when treated with cetuximab compared to patients treated with chemotherapy alone, whereas patients with low miR-31-3p expression had similar clinical outcomes for both study treatment arms.

Although we could not demonstrate the predictivity of miR-31-3p (homogeneity test p-value p=0.16), this is likely due to the low number of patients and lack of maturity of the data (52% censored patients for progression free survival and 82% for overall survival). The power of this study to demonstrate predictivity, computed a posteriori, was only 65.3%. [21] Similarly, the absence of demonstration of a prognostic effect of miR-31-3p on overall survival in the cetuximab-treated population is likely to be related to the limited number of events.

We failed to observe a clear association between miR-31-3p expression and response to cetuximab. This could be due to several reasons including the high objective response rate observed both in the chemotherapy alone (42/71 59%) and cetuximab (54/78 69%) arms suggesting that cetuximab impact on the tumor response was relatively marginal, limiting the capacity to demonstrate an interaction between miR-31-3p expression and response in this setting.

While analysis of miR-31-3p expression and its association to clinical outcomes did not enable the ability to identify a patient subgroup that benefited from the addition of cetuximab to chemotherapy, this needs to be interpreted in the context of the results of the New EPOC study in which the addition of cetuximab to chemotherapy produced a detrimental effect. However, our results show that the poorer outcomes associated with the addition of cetuximab to chemotherapy were limited to patients who had middle to high miR-31-3p expression and that this detrimental effect was not observed in patients with low miR-31-3p expression levels. As such, the predictive value of miR-31-3p expression is consistent with the primary outcome of the New EPOC study.

The potential interest of miR-31-3p expression as a predictive biomarker of the response to anti-EGFR therapy in metastatic colorectal cancer has been previously reported in several papers. [19, 20] Preliminary results of studies assessing the association with miR-31-3p expression and therapeutic benefit from anti-EGFR therapy have also been reported for the PICCOLO and FIRE-3 studies. [22, 23] The present analysis was performed prior to the determination of the optimal cut-off value for miR-31-3p that has recently been reported based on the analysis of tumors samples from patients enrolled in the FIRE-3 trial. [23] As a result, the present study in which the patient population was separated into tertiles represents an initial exploration of potential cut-off points for miR-31-3p expression as it relates to differential outcomes. Although the specific context and results of the study as well as the lack of maturity of the data, prevent the generalization of the conclusions to other clinical settings, we believe the present study contributes to the accumulation of data supporting the clinical and biological rationale of miR-31-3p expression as a predictive biomarker of response to anti-EGFR therapy in metastatic colorectal cancer.

The loss of correlation of miR-31-3p expression level for the cetuximab group between primary tumors, which were collected prior to any treatment, and liver metastasis, which were collected after treatment, suggests that treatment with cetuximab may alter the expression of this microRNA. This finding suggests the regulation of miR-31-3p expression as a part of the EGFR pathway and is consistent with a previous report where maturation of mir31 in the miR-31-5p form was found to be regulated via EGFR in a tumor hypoxia context. [24] It is also important to note that the selection of miR-31-3p for the present study was done following a broad screening of 1,145 miRNAs, [19] and as a result of previous findings that pre-mir-31 leads to maturation of the highly correlated mature forms of mir-31, miR-31-3p and miR-31-5p. [20] Despite this high correlation between the two mature forms of mir-31, prediction of response to anti-EGFR therapy differs between miR-31-3p and miR-31-5p, suggesting the existence of fine regulation mechanisms involving specifically miR-31-3p or genes targeted by miR-31-3p, beyond regulation of mir-31 expression. [18]

In summary, analysis of miR-31-3p expression enabled the identification of a subgroup of patients with operable colorectal liver metastases who have a poorer outcome when treated with chemotherapy plus cetuximab. While the mechanism of this differential effect relative to miR-31-3p expression requires investigation, these data support a biological explanation for the detrimental effect observed with the use of cetuximab in patients in the New EPOC study, rather than, for example, inadequate surgery being responsible. Further work is ongoing to validate the use of this promising predictive biomarker in advanced colorectal cancer.

## MATERIALS AND METHODS

### Patients and samples

The New EPOC trial has previously been reported [5] and the full protocol can be found on the Southampton Clinical Trials Unit website (www.ctu.soton.ac.uk). Patients with resectable or suboptimally resectable liver metastases from colorectal cancer were randomly allocated to two treatment arms; chemotherapy (oxaliplatin/irinotecan plus fluorouracil) with or without cetuximab given before and after liver resection. The study was closed by the Trial Steering Committee on advice from the Data Monitoring and Ethics Committee (DMEC) in November 2012 when the protocol defined futility criteria were met (the lower limit of the 95% Confidence Interval (CI) for progression free survival HR was >1). [25]

Primary tumor and liver metastasis samples collected from patients enrolled in the New EPOC trial were analyzed for the present study. As per the New EPOC study protocol, primary tumor samples were collected at baseline and samples for liver metastases were collected following neoadjuvant treatment and liver resection. Primary tumors from 167 of the 236 patients in the original primary analysis population [5] were available for extended RAS mutational testing and miR-31-3p expression in the current analysis. The modified intention to treat (mITT) population was defined as those patients included in the original New EPOC primary analysis population (e.g. those who were KRAS wild-type for codons 12, 13 and 61 at trial entry) in whom further testing did not demonstrate any RAS mutation. This resulted in a mITT population comprised of 149 patients. Liver metastasis samples were available for 147 patients enrolled in the original New EPOC study with paired samples of both primary tumor and metastasis available for a total of 94 included in the mITT population.

### Mutational analyses

Primary tumors and metastases analyzed for the present study were sequenced for KRAS codons 12, 13, 61, 117 and 146, NRAS codons 12, 13, 61, 117 146, and BRAF codon 600 using Sequencing By Synthesis (SBS) on a MiSeq platform (Illumina, San Diego, California). Mutations were identified with respect to two known non-mutated controls for all mutations considered and the three positions of the codon were analyzed when needed. A 99% confidence interval for the minor allele frequency was calculated, taking into account the total number of bases read for a nucleotide in a particular codon and the number of “new” bases read for the same nucleotide of the same codon. Mutations were considered actual results when the same base was identified regarding the 2 controls. A frequency below 5% was considered as non-mutated. Samples which had indeterminate results were resequenced using Ion Torrent™ Technology (Thermo Fisher Scientific, Carlsbad, CA). For this analysis libraries were prepared according the manufacturer’s instruction (Ion AmpliSeq™ DNA and RNA Library Preparation Rev.B, Ion Torrent, Thermo Fisher Scientific, Carlsbad, CA). Samples were amplified with the Ion AmpliSeq™ Cancer HotSpot Panel v2 and the concentration of each library was determined with Experion™ Automated Electrophoresis system (Bio-Rad Laboratories Inc., Hercules, CA). Emulsion PCR was done according to the manufacturer’s user guide (Ion PGM™ Template OT2 200 Kit Revision A.0). Sequencing was performed using two Ion 318TM Chip V2 on an Ion PGM System™. Data analysis, including alignment to the hg19 human reference genome and variant calling, was done using the Torrent Suite Software v4.0. Alignments were visually verified and annotated with the ALAMUT software v2.2 (Interactive Biosoftware, Rouen, France).

### MiR-31-3p expression analyses

A pathologist reviewed all samples and the tumor area was marked for subsequent macrodissection. Only samples with more than 20% tumor cell content were selected for nucleic acid extraction. For each tumor sample, 5 FFPE slides of 5μm thickness were scratched in the tumor area and total RNA was extracted using the FFPE miRNeasy extraction kit (Qiagen, Hilden, Germany) according to the manufacturer’s instructions. Specific quantification of expression level of miRNA hsa-miR-31-3p was performed on retrotranscribed RNA using specific TaqMan pre-designed assays on ABI7900HT Real-Time PCR System. Expression levels were normalized to a reference miRNA using the ΔΔCt method.

### Statistical analysis

The primary endpoint was progression free survival defined as the time from randomization to recurrence, disease progression, or death, whichever occurred first. Secondary endpoints included overall survival (time from randomization to death; patients still alive at the date of last follow-up were censored) and preoperative response (using Response Evaluation Criteria in Solid Tumors (RECIST) version 1.0). All analyses reported here include data up to November 1, 2012 to match the time frame presented in the interim report to the DMEC.

Analyses were performed for the modified Intent-to-Treat (mITT) population which consisted of 149 KRAS WT patients whose RAS status was either WT or indeterminate. Patients whose tumors were confirmed to be KRAS or RAS mutated were excluded from the analysis. In line with the study’s pre-specified statistical analysis plan, patients were divided into three subgroups defined as either low, medium (mid) or high miR-31-3p expression levels based on tertiles of miR-31-3p expression distribution. Progression free survival and overall survival comparisons according to miR-31-3p expression tertile and between each treatment arm were performed using Kaplan-Meier (KM) methodology and two-sided log-rank test. Objective Response Rate according to tertiles and across treatment arms were compared using Fisher exact test, considering non-evaluable patients as non-responders. Because similar results were observed in the mid and high tertile subgroups (Supplementary Figure 1), these subgroups were merged into a single “high” miR-31-3p expression subgroup and subsequent analyses compared the “low” miR-31-3p expression subgroup (low tertile) with the “high” subgroup (mid + high tertiles). Impact of covariates on progression free survival or overall survival (i.e. age, gender, ECOG status, tumor location, BRAF mutational status, chemotherapy backbone, number of liver metastases) was first analyzed in separate univariate Cox models and then in a multivariate stepwise Cox model. Potential predictors with univariate p-value below 0.20 were selected to enter in the model and were kept in the multivariate model if their multivariate p-value was below 0.10. MiR-31-3p predictivity was explored in this multivariate model using an interaction term of treatment with miR-31-3p subgroup. Expression levels of miR-31-3p in LM vs PT were compared using the log of miR-31-3p expression fold and a paired Student t test. A Pearson test was performed to evaluate correlation of miR-31-3p expression in primary tumors and their paired liver metastases. Two-way analysis of variance was used to test paired primary tumor-liver metastases and treatment effects on miR-31-3p expression.

## SUPPLEMENTARY MATERIALS FIGURE


