# Supplementary material for: Association between miR-31-3p expression and cetuximab efficacy in patients with KRAS wild-type metastatic colorectal cancer: a post-hoc analysis of the New EPOC trial
Source: Oncotarget. 2017 Sep 27;8(55):93856–66. doi: 10.18632/oncotarget.21291 (PMC5706840; doi:10.18632/oncotarget.21291)
Supplement: Supplementary file 1 [file oncotarget-08-93856-s001.pdf]

# Association between miR-31-3p expression and cetuximab efficacy in patients with KRAS wild-type metastatic colorectal cancer: a post-hoc analysis of the New EPOC trial

## SUPPLEMENTARY MATERIALS

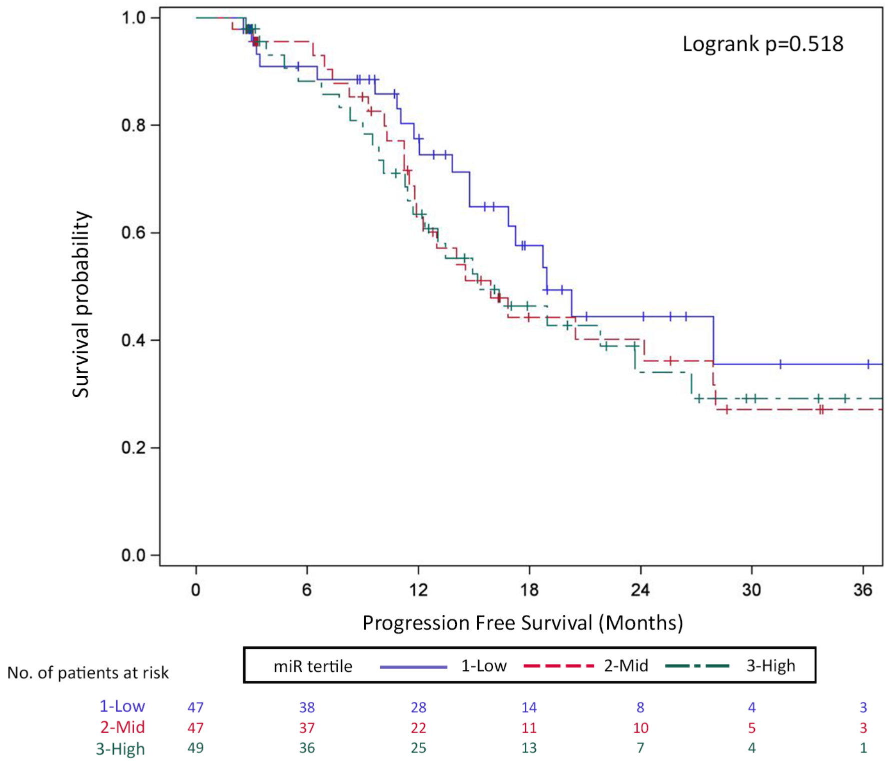


**Supplementary Figure 1: Kaplan-Meier curves of progression-free survival according to miR-31-3p expression level groups (mITT population).**
